# Development of a Stable Oxygen Sensor Using a 761 nm DFB Laser and Multi-Pass Absorption Spectroscopy for Field Measurements

**DOI:** 10.3390/s23094274

**Published:** 2023-04-25

**Authors:** Jvqiang Chang, Qixin He, Mengxin Li

**Affiliations:** MoE Key Lab of Luminescence and Optical Information, Beijing Jiaotong University, No. 3 Shangyuancun, Beijing 100044, China

**Keywords:** optical sensing, oxygen sensor, absorption spectroscopy, WMS-2*f*/1*f*

## Abstract

An optical sensor system based on wavelength modulation spectroscopy (WMS) was developed for atmospheric oxygen (O_2_) detection. A distributed feedback (DFB) laser with butterfly packaging was used to target the O_2_ absorption line at 760.89 nm. A compact multi-pass gas cell was employed to increase the effective absorption length to 3.3 m. To ensure the stability and anti-interference capability of the sensor in field measurements, the optical module was fabricated with isolation of ambient light and vibration design. A 1*f* normalized 2*f* WMS (WMS-2*f*/1*f*) technique was adopted to reduce the effect of laser power drift. In addition, a LabVIEW-based dual-channel lock-in amplifier was developed for harmonic detection, which significantly reduced the sensor volume and cost. The detailed detection principle was described, and a theoretical model was established to verify the effectiveness of the technique. Experiments were carried out to obtain the device’s sensing performances. An Allan deviation analysis yielded a minimum detection limit of 0.054% for 1 s integration time that can be further improved to 0.009% at ~60 s. Finally, the reliability and anti-interference capability of the sensor system were verified by the atmospheric O_2_ monitoring.

## 1. Introduction

Oxygen (O_2_) is one of the most common gases and plays an indispensable role in people’s daily lives, as well as in industrial production. For example, O_2_ in indoor and outdoor environments is closely related to people’s breathing. Low oxygen concentration in an environment can cause weakness, dizziness, and even loss of consciousness. Low oxygen levels can result from activity in certain workplace settings, such as mining or welding, where heavy machinery can deplete the oxygen supply. OSHA (the Occupational Safety and Health Administration) specifies that a hazardous atmosphere may include one where the O_2_ concentration is below 19.5% or above 23.5%, so O_2_ concentration monitoring is important for ensuring safety in confined spaces, such as coal mines and tunnels [1,2]. Thus, analyzing the oxygen levels in these settings is essential to ensuring the safety of individuals working in such environments. In metallurgy, energy, and other industrial fields, O_2_ is used as an accelerant that directly affects the combustion process. Measuring the O_2_ concentration can effectively improve the performance of the engine and reduce the emission of pollutants [3,4]. Furthermore, measuring O_2_ concentration in natural environments, including forests and oceans, can help identify changes in ecosystems, which may indicate pollution or other underlying issues.

O_2_ concentration detection can be achieved through various methods, including the electrochemical method [5], gas chromatography [6], and optical methods. In recent years, optical-based measurement methods have garnered increased attention from researchers. Compared with traditional O_2_ detection methods, such as electrochemical method, gas chromatography, optical methods including optical absorption spectroscopy, quartz-enhanced photoacoustic spectroscopy (QEPAS) [7,8], and magnetic rotation spectroscopy (MRS) [9,10,11] have the advantage of fast response, non-contact measurement, and long life, making these methods suitable for online O_2_ monitoring. Tunable diode laser absorption spectroscopy (TDLAS) is an optical absorption spectroscopy employed in many fields due to its low cost, simple structure, and high sensitivity [12,13,14]. Some examples of O_2_ concentration measurement using TDLAS include the following. In 2009, Zhang S A et al. used a DFB laser as a light source to detect O_2_ with an absorption line near 760 nm by TDLAS combined with wavelength modulation spectroscopy (TDLAS-WMS), realizing a detection accuracy of 1000 ppmv [15]. In 2017, an O_2_ sensor system was proposed by Zhou X et al. with a cubic diffusion integral cavity as the gas absorption cell to increase the absorption optical path length, and a detection limit of 350 ppmv was achieved by the measurement of O_2_ absorption line at 764.38 nm [16]. In 2022, Luo Q W et al. proposed an effective interference fringe suppression method based on machine learning, which has been applied to the measurement of O_2_ concentration in medical bottles to achieve an average absolute inversion error of 0.57% [17].

Compared with directly using the second harmonic signal (2*f*) for concentration inversion, 1*f*-normalized 2*f*-wavelength modulation spectroscopy (WMS-2*f*/1*f*) has stronger anti-interference to the fluctuations of light intensity and adapts better to harsh environments. The WMS-2*f*/1*f* technology was developed by Rieker G B and co-workers [18] and was adopted by many other researchers [19,20]. In 2014, Neethu S et al. established an experimental set-up and measured O_2_ using three methodologies: (i) using only the second harmonic; (ii) using the second and fourth harmonics; and (iii) using the first and second harmonics. The advantages of the third technique in resisting laser intensity variations were proved [21]. Li C G et al. designed a dense multi-pass gas cell with an optical path of 54.6 m and raised the minimum detection limit (MDL) of ethane to 239 pptv using WMS-2*f*/1*f* technology in 2016 [22]. In 2017, He Q X et al. designed a near-infrared methane detection system with a hollow-core photonic crystal fiber (HC-PCF) as a gas absorption cell, which achieved an MDL of 8.7 ppmv in an average time of 0.1 s [23]. In 2019, Guo X Q et al. developed a portable ammonia sensor, which used the absorption line of 1500 nm to measure the concentration of ammonia, and the MDL could reach 0.16 ppmv at 184 s [24]. More recently, in 2022, Zhang L W et al. introduced a device for detecting carbon monoxide in the exhaust pipeline of a furnace based on WMS-2*f*/1*f* technology and achieved an MDL of 1.299 ppmv [25]. However, most of the above systems were built on optical platforms, and the experiments were conducted in the laboratory. In addition, commercial lock-in amplifiers were used in these systems for signal extraction, which was difficult to integrate due to the large volume.

Although some studies have reported the use of WMS for O_2_ detection, to the best of our knowledge, few studies have employed WMS-2*f*/1*f* for this purpose. In this study, we reported on developing an O_2_ sensor system based on WMS-2*f*/1*f* technology using a 761 nm DFB laser. A compact multi-pass gas cell with a 3.3 m optical path length was used, and a dual-channel digital orthogonal lock-in amplifier was designed based on LabVIEW to extract the 1*f* signal and the 2*f* signal. The theory and simulation of WMS-2*f*/1*f* technology were introduced first, then the design details of the sensor system were described. The sensor performance, including linearity, stability, and detection limits, was evaluated by laboratory experiments. Finally, field experiments were conducted to verify the reliability and anti-interference ability of the sensor system.

## 2. Theory and Simulation

### 2.1. Theory of WMS-2f/1f Technology

According to the Beer-Lambert law, when a laser passes through a target gas with an optical path L, the light intensity will decrease, which can be expressed as:

(1) $$I=I_{0}exp\left[-\alpha \left(v\right)CL\right]$$

where $I_{0}$ and I indicate the incident and outgoing light intensity, respectively; C is the concentration of the gas; $\alpha \left(v\right)$ is the absorption coefficient of the corresponding gas: $\alpha \left(v\right)=NS\left(T\right)\varphi \left(v\right)$; $S\left(T\right)$ ($cm\cdot mol^{-1}$) is the line strength; $\varphi \left(v\right)$ (cm) is the line shape function; $N=P/\left(k\cdot T\right)$ is the molecular density of the gas, where P $\left(atm\right)$ is the total pressure, k is the Boltzmann constant, and T $\left(K\right)$ is the temperature. Considering that collisional broadening plays a major role in the full-width at half-maximum (FWHM) at normal temperature and pressure, Lorentz lineshape is selected as the line shape function in this paper.

As $\alpha \left(v\right)L\ll 1$, Equation (1) can be transformed into:

(2) $$I\approx I_{0}\left(1-\alpha \left(v\right)CL\right)$$

In wavelength modulation spectroscopy (WMS) technology, a low-frequency triangular driver signal and a high-frequency sinusoidal driver signal were generated to scan across the selected absorption line and suppress the background noise. After modulation, the frequency and light intensity can be expressed as [18,26]:

(3) $$v\left(t\right)=v_{0}+acos\left(\omega t+\psi \right)$$

(4) $$I_{0}\left(t\right)=\bar{I_{0}}\left(1+i_{1}cos\left(\omega t+\psi_{1}\right)+\overset{\infty }{\underset{m=2}{\sum }}i_{m}cos\left(m\omega t+\psi_{m}\right)\right)$$

where $v_{0}$ is the center frequency of the laser; a is the frequency modulation depth; ψ is the phase of frequency modulation$;\;I_{0}\left(t\right)$ represents the light intensity after modulation; $\;I_{0}$ is average laser intensity over modulation period; and $i_{1}$ and $i_{m}$ are the linear and nonlinear light intensity modulation coefficients. Generally, m is only 2, and the modulation coefficients of higher harmonics are small and can be ignored. The $\psi_{1}$ and $\psi_{m}$ represent the phase differences of the output light intensity before and after modulation; and $\omega =2\pi f_{m}$, $f_{m}$ represents the modulation frequency of the high-frequency sinusoidal signal.

After Fourier expansion of the transmissivity $\tau \left(v\right)$, the following can be obtained:

(5) $$\tau \left(v\right)={\left(\frac{I}{I_{0}}\right)}_{v}=\overset{\infty }{\underset{k=0}{\sum }}H_{k}cos\left(k\omega t\right)$$

Combined with Equation (5), the k-th harmonic coefficient can be obtained:

(6) $$H_{0}=-\frac{NS\left(T\right)CL}{2\pi }\int_{-\pi }^{\pi }\varphi \left(v\right)d\omega t$$

When k≠0:

(7) $$H_{k}=-\frac{NS\left(T\right)CL}{\pi }\int_{-\pi }^{\pi }\varphi \left(v\right)cos\left(k\omega t\right)d\omega t$$

It can be seen that the harmonic components are in direct proportion to the gas concentration. The $X_{2f}\;$ and $Y_{2f}$ components of the 2*f* signal can be obtained by an orthogonal lock-in amplifier (multiplying the absorbed signal with the cosine reference signal $cos\left(2\omega t\right)$ and the sine reference signal $sin\left(2\omega t\right)$, respectively, and then pass the low pass filter):

(8) $$X_{2f}=\frac{G\bar{I_{0}}}{2}\left(H_{2}+\frac{i_{1}}{2}\left(H_{1}+H_{3}\right)cos\psi_{1}+i_{2}\left(H_{0}+\frac{H_{4}}{2}\right)cos\psi_{2}\right)$$

(9) $$Y_{2f}=-\frac{G\bar{I_{0}}}{2}\left(\frac{i_{1}}{2}\left(H_{1}-H_{3}\right)sin\psi_{1}+i_{2}\left(H_{0}-\frac{H_{4}}{2}\right)sin\psi_{2}\right)$$

Similarly, the $X_{1f}$ and $Y_{1f}$ can be expressed as follows:

(10) $$X_{1f}=\frac{G\bar{I_{0}}}{2}\left(H_{1}+i_{1}\left(H_{0}+\frac{H_{2}}{2}\right)cos\psi_{1}+\frac{i_{2}}{2}\left(H_{1}+H_{3}\right)cos\psi_{2}\right)$$

(11) $$Y_{1f}=-\frac{G\bar{I_{0}}}{2}\left(i_{1}\left(H_{0}-\frac{H_{2}}{2}\right)sin\psi_{1}+\frac{i_{2}}{2}\left(H_{1}-H_{3}\right)sin\psi_{2}\right)$$

where G is the conversion coefficient of photodetector, and the 1*f* signal can be obtained:

$$R_{1f}=\sqrt{X_{1}^{2}+Y_{1}^{2}}$$

The expression of the WMS-2*f*/1*f* after deducting the background signal is as follows:

(12) $$R_{2f/1f}=\sqrt{{\left[\left(\frac{X_{2f}}{R_{1f}}\right)-{\left(\frac{X_{2f}}{R_{1f}}\right)}_{bgr}\right]}^{2}+{\left[\left(\frac{Y_{2f}}{R_{1f}}\right)-{\left(\frac{Y_{2f}}{R_{1f}}\right)}_{bgr}\right]}^{2}}$$

Since the harmonic expansion of Lorentz lineshape has even harmonic characteristics, the amplitude of the odd harmonic component is 0 at the center frequency, and low concentration gases, $H_{0}$, $H_{2}$ and $H_{4}$, are both far less than 1. Therefore, at the center frequency, the Equation (12) can be simplified as:

(13) $$R_{2f/1f}\approx \frac{\frac{G\bar{I_{0}}}{2}H_{2}}{\frac{G\bar{I_{0}}}{2}i_{1}}=\frac{H_{2}}{i_{1}}$$

As can be concluded from Equation (13), the WMS-2*f*/1*f* method can effectively avoid the interference of laser intensity fluctuations, temperature drift, and pressure drift, thus improving the anti-interference of the system.

### 2.2. Selection of O_2_ Absorption Line

**Figure 1:**
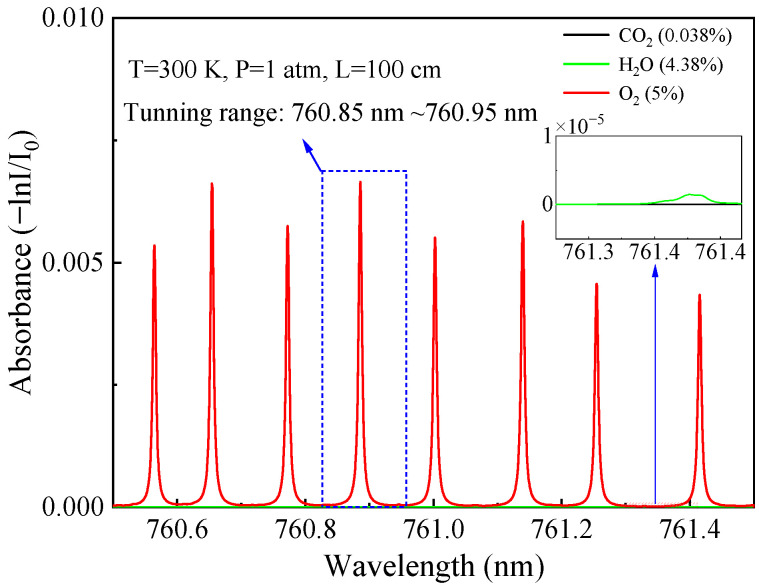
HITRAN based absorption spectra of O_2_ (5%), H_2_O (4.38%), and CO_2_ (0.038%) in a spectral range from 760.5 nm to 761.5 nm for a 100 cm path length at a pressure of 1 atm. O_2_, H_2_O, and CO_2_ lines are shown in red, green, and black, respectively.

The main factors to be considered in the selection of an absorption line are the line strength, selectivity, and system cost [27]. O_2_ has a relatively strong absorption band near 760 nm. A spectral simulation of 5% O_2_ in this spectral range was conducted according to the HITRAN 2020 database [28] under a condition of 300 K, 1 atm, and 100 cm absorption length as depicted in Figure 1. Considering the sensor system should work normally in an atmospheric environment, the absorption of 0.038% carbon dioxide and 4.38% water vapor were also simulated. It can be seen that H_2_O and CO_2_ in ambient air produce little interference to the O_2_ detection in this wavelength, and the absorption line at 760.89 nm was selected as the optimal absorption line due to its strong line strength.

A DFB laser emitting at 761 nm was chosen as the laser light source. The tuning range of the laser was set to be 760.85 nm to 760.95 nm to scan across the optimal absorption line. According to the laser wavelength tuning experiment, the temperature and current tuning coefficient of the laser were 0.014 nm/mA and 0.06 nm/°C, respectively. In the experiment, the laser operating temperature was set to 40 °C, and the driving current was swept from 27 mA to 40 mA.

### 2.3. Simulation

In order to verify the feasibility of WMS-2*f*/1*f* technology and optimize the parameters of the system, a simulation model of the O_2_ sensor system was established by the MATLAB Simulink platform based on the WMS-2*f*/1*f* theory and the parameters of the selected absorption line. Next, the influence of concentration and modulation coefficients on the sensor output signals were simulated by the model.

In WMS, the modulation coefficient is defined as $m=\frac{a}{\Delta v_{c}/2}$, where a is the frequency modulation depth, and $\Delta v_{c}/2$ is the half-width at half-maximum (HWHM) of the gas sample absorption line. At the central frequency, the amplitude of the second harmonic coefficient is:

(14) $$H_{2}\left(0,m\right)=-\frac{NCS\left(T\right)L}{\pi \Delta v_{c}}\left[\frac{4}{m^{2}}-\frac{2\left(2+m^{2}\right)}{m^{2}\sqrt{1+m^{2}}}\right]$$

**Figure 2:**
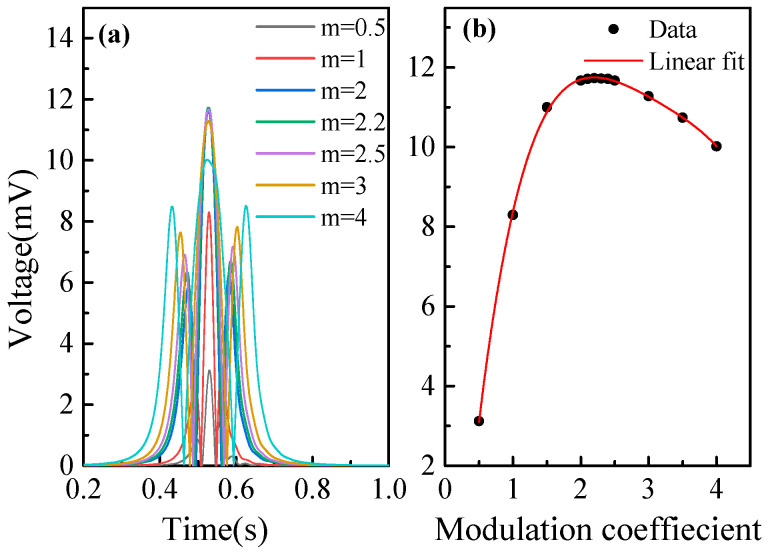
(**a**) Simulated 2*f* signals at different modulation coefficients. (**b**) Simulated amplitude of the WMS-2*f* signals at different modulation coefficients.

From Equation (14), it can be seen that the modulation coefficients (m) directly affect the amplitude of the second harmonic ($H_{2}\left(0,m\right)$). Using $\frac{dH_{2}\left(0,m\right)}{dm}=0$, it can be calculated that the largest second harmonic amplitude is at m≈2.2. In the simulation, the concentration was set to 1%, and m was changed from 0.5 to 4. The waveform and amplitude of 2*f* signals under different modulation coefficients were simulated as depicted in Figure 2a,b. The largest 2*f* amplitude was obtained at m≈2.2, which is consistent with the theoretical value.

**Figure 3:**
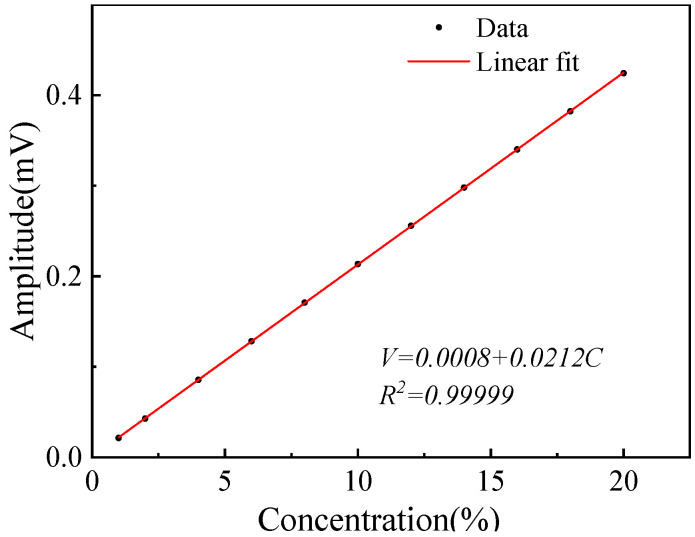
Simulated amplitude of the WMS-*2f*/*1f* signals at different concentrations.

The relationship between the 1*f* normalized 2*f* (WMS-2*f*/1*f*) signal and the O_2_ concentration was simulated under the condition of optimal modulation coefficients. The WMS-2*f*/1*f* signals with a concentration of 1~20% were obtained by the simulation model, then the relationship was fitted as shown in Figure 3. The correlation coefficient is >0.999, which indicates this method has good linearity.

## 3. System Design

### 3.1. System Structure

**Figure 4:**
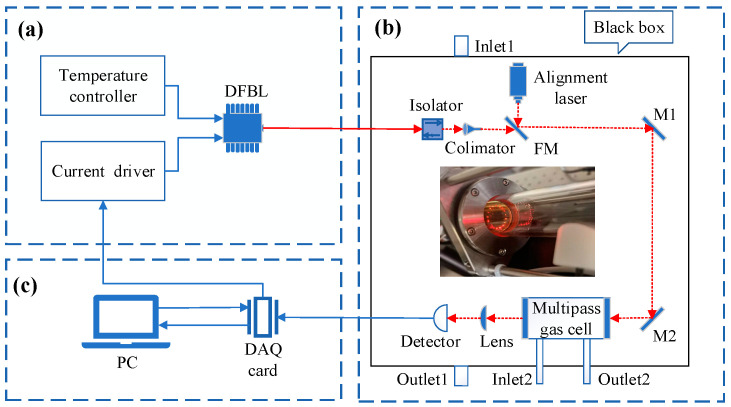
The schematic diagram of the O_2_ detection system. (**a**) Light source module; (**b**) optical module; (**c**) data processing module. DFBL: distributed feedback laser, FM: flip mirror.

The architecture of the O_2_ sensor system, depicted in Figure 4, is mainly composed of three modules: light source module, optical path transmission module, and signal processing module. In the light source module, the temperature of the DFB laser (Nanoplus, nominal power is ~5 mW) was stabilized by a temperature controller (Thorlabs, TED200C, Perth Amboy, NJ, USA). A low-frequency triangular signal (2 Hz) and a high-frequency sinusoidal signal (2 kHz) were generated and superimposed to a current driver (Thorlabs, LDC210C) to modulate the laser current. The DFB laser was connected to an optical isolator (Thorlabs, IO-F-780APC) to avoid the interference of the reflected light to the light source. Then the emitting light was collimated by a collimator (Thorlabs, F230APC-780). A flip mirror and two reflective mirrors (M1 and M2) were used to induce the laser and the reference light into a compact multi-pass gas cell (URAY-Herriott Cell). With 16 times reflection in the gas cell, an effective optical path of 3.3 m was obtained. The optical signal with O_2_ concentration information emitted from the multi-pass gas cell was focused to a photoelectric detector (Thorlabs, PDA100A2) and then processed in a computer with a DAQ card (National Instrument, USB-6211, Austin, TX, USA) and LabVIEW software platform. The dimensions of the optical path transmission module are 47 × 32 × 24 (length × width × height), the light source module has dimensions of 31 × 26 × 11 (length × width × height), and the signal processing module has dimensions of 33 × 23 × 3 (length × width × height). By selecting lasers and detectors of different wavelengths, it is possible to detect various other gases.

To achieve reliable and accurate ambient O_2_ detection in field environments, the sensor design incorporates the following measures. To reduce the effect of vibration in the environment, a switchable gain Silicon amplified detector with a large active area of 75.4 mm^2^ was selected. To reduce the interference of ambient O_2_ and the environmental light, the optical module was sealed and covered with black shade papers. Before measurement, the optical module was purged with pure nitrogen through Inlet 1, while air was discharged through Outlet 1. A vacuum pump (Kamoer, HLVP15, Shenzhen, China) was connected to Outlet 2 to pump gas into the gas cell, and a drying tube was connected to Inlet 2 to remove moisture from the gas sample.

### 3.2. LabVIEW-Based Data Processing and Signal Generation Platform

**Figure 5:**
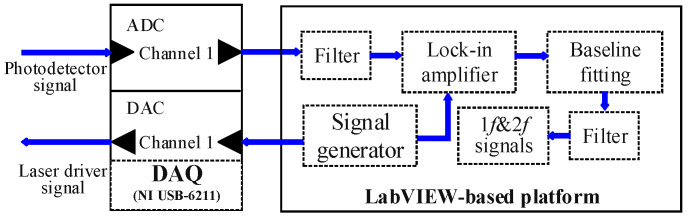
Function diagram of the LabVIEW-based data processing and signal generation platform. ADC: analog-to-digital converter; DAC: digital-to-analog converter.

A LabVIEW-based platform was developed for data processing and laser driver signal generation. The function diagram of the platform was shown in Figure 5. The analog electronic signal generated by the detector was sent to the laptop through the analog-to-digital converter (ADC) module of the DAQ card. Then the collected digital signal was processed in the LabVIEW platform including harmonic extraction, filtering, and background signal fitting. The third order polynomial baseline fitting was conducted to reduce the influence of light intensity variation and waveform distortion. A dual-channel digital lock-in amplifier was developed for the extraction of 1*f* signal and 2*f* signal. Peak-to-peak amplitude of 2*f* signal was chosen and normalized by 1*f* signal to calculate the concentration. The signal generator module in LabVIEW was designed to generate a driver signal for laser current modulation. Simultaneously, the module produced a reference signal with the same frequency and phase as the driver signal, which serves for the harmonic extraction of the lock-in amplifier.

## 4. Sensor Performance

### 4.1. Modulation Amplitude Optimization

**Figure 6:**
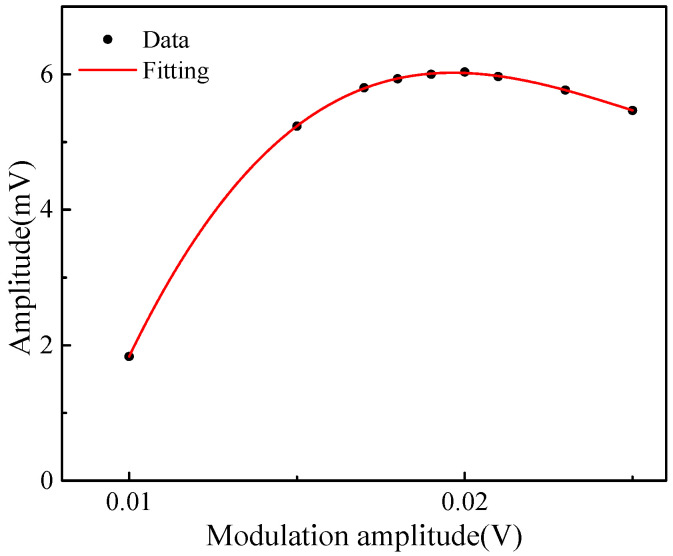
Measured amplitude of the 2*f* signal versus the modulation amplitude.

The experiments were conducted at pressure of 1 atm and temperature of 23 °C. The frequency of the triangular signal was 2 Hz. In order to scan across the selected absorption line at 760.89 nm, the amplitude of the triangular signal was set to 0.15 V. In order to determine the optimum modulation amplitude of the sensor system, experiments on different modulation amplitudes were conducted. Measured amplitude of the 2*f* signal versus the modulation amplitude were shown in Figure 6. It can be seen that the variation trend of 2*f* signal amplitude with modulation amplitude is consistent with the theoretical, and the maximum amplitude is ∼0.02 V. Thus, 0.02 V was selected as modulation amplitude.

### 4.2. Calibration and Fitting

**Figure 7:**
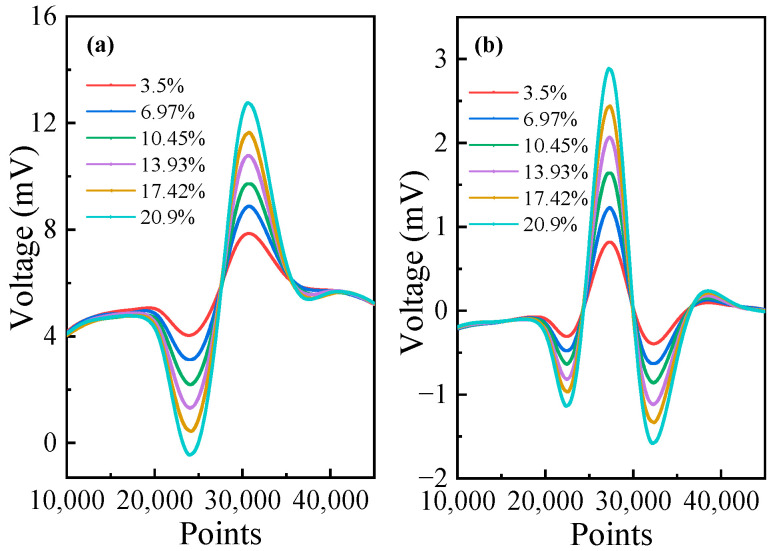
The 1*f* and 2*f* signals at different O_2_ concentration levels. (**a**) 1*f* signals, (**b**) 2*f* signals.

O_2_ samples with different concentrations from 3.5% to 20.9% were prepared by mixing pure N_2_ with pure O_2_. The prepared gas samples were pumped into the multi-pass gas cell, and the extracted 1*f* and 2*f* signals were shown in Figure 7. As for the 3.5% O_2_ sample, the amplitude and noise level of 2*f* signals were roughly 1.206 mV and 2.02 µV. The SNR was calculated to be 27.06 dB. It can be seen that the maximum value of the 2*f* signal increases with the concentration levels, while the mean value of the 1*f* signal remains constant. Hence, the mean value of the 1*f* signal was used to normalize the 2*f* signal in the experiment to reduce the effect of laser power variations.

**Figure 8:**
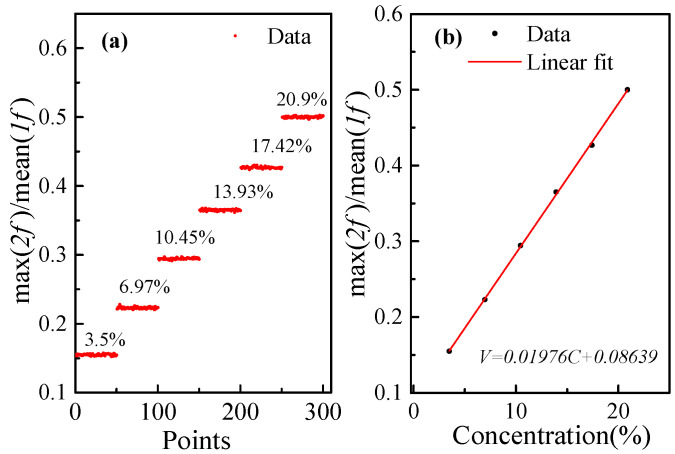
(**a**) The 2*f*/1*f* signal amplitudes at different O_2_ concentration levels. (**b**) The linear fitting curve.

When the concentration in the gas cell reaches a stable level, the ratio of maximum (2*f*)/mean (1*f*) was recorded for 50 s at a sampling frequency of 1 Hz, as shown in Figure 8a. Figure 8b shows the relationship between the ratio of maximum (2*f*)/mean (1*f*) and the concentration. After averaging the sampling data at each concentration, linear fitting was carried out, and the relationship was calculated as follows:

(15) $$V_{2f/1f}=0.01976C+0.08639$$

The square of the correlation coefficient of the fitting curve is $R^{2}=0.9995$.

### 4.3. Response Time and Stability

**Figure 9:**
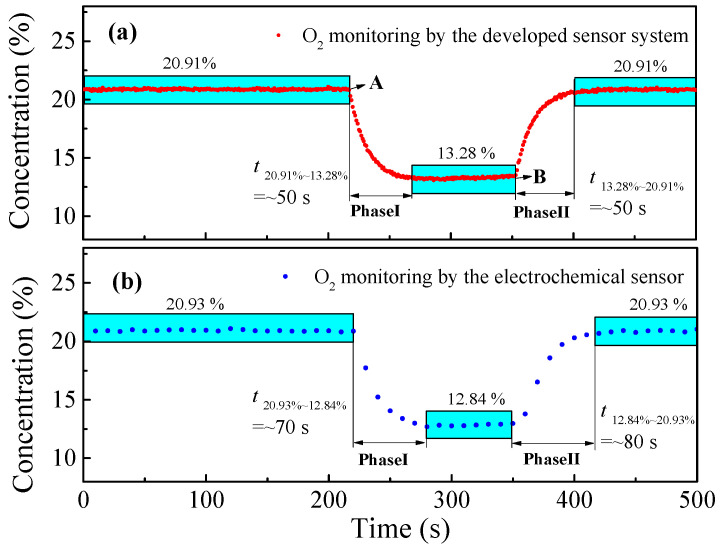
Response time measurement result by varying O_2_ concentration levels. (**a**) Measured by the designed sensor. (**b**) Measured by the electrochemical sensor.

The response time mainly depends on the gas cell volume and the gas flow rate. In order to determine the response time of the designed sensor system, a pump was connected to the outlet of the 50 mL gas cell, and the pumping rate was set to 400 mL/min. The experiment was divided into two phases: in phase I, the O_2_ concentration was changed from 20.9% to 13.3% starting from point A; and in phase II, the O_2_ concentration was changed from 13.3% to 20.9% starting from point B. The variation trend of the measured concentration was shown in the Figure 9a. At the same time, a commercial electrochemical O_2_ detector (SAFEGAS, SKY6000, Shanghai, China) was connected in series with the gas cell for comparison, and the results are shown in Figure 9b. The concentration curve indicated that the designed sensor response time is ∼50 s for phase I and phase II, which is shorter than the commercial electrochemical O_2_ detector. The trends of the two sensors were consistent, a finding which also verifies the effectiveness of the designed sensor system.

**Figure 10:**
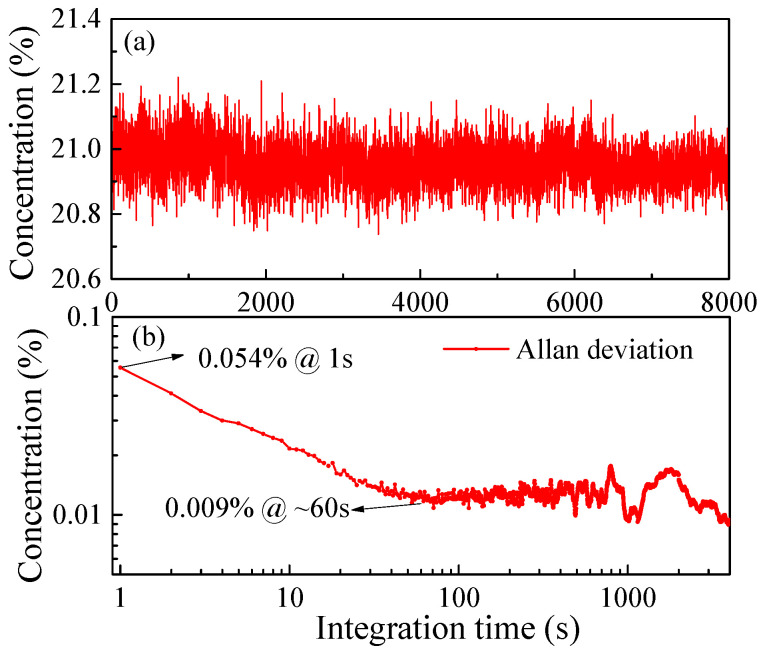
(**a**) Stability test result. (**b**) Allan variance analysis for the sensor system.

In order to test the stability of the system, measurements of an O_2_ sample with a concentration of 20.9% over time periods of ~2.2 h were performed. An amount of 8000 measured points was obtained as shown in Figure 10a. Allan variance analysis was performed to evaluate the MDL of the system. As shown in Figure 10b, the MDL is 0.054% under 1 s integration time. When the integration time reaches ~60 s, the optimal MDL is 0.009%.

## 5. Atmospheric O_2_ Detection

**Figure 11:**
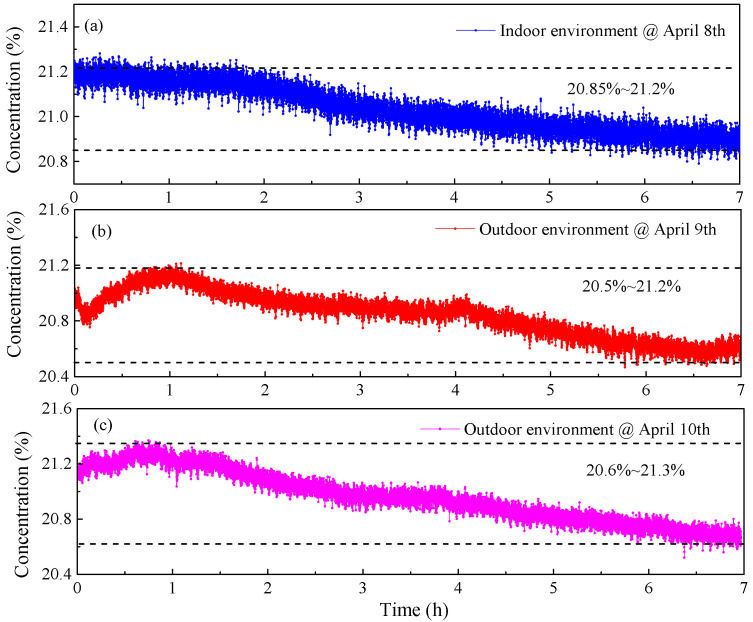
(**a**) Measured concentrations of O_2_ in indoor environment. (**b**) Measured concentrations of O_2_ in outdoor environment on 9 April. (**c**) Measured concentrations of O_2_ in outdoor environment on 10 April.

The designed sensor system was also evaluated for the detection of atmospheric O_2_ at the Beijing Jiaotong University. The optical module was sealed in a black box, which can effectively avoid the influence of ambient light. Indoor measurements were conducted in the Optoelectronic Science and Technology Laboratory. The measurements were conducted on 8 April, lasting for a total of seven hours, from 11 a.m. to 6 p.m. The summarized results of the measurements can be found in Figure 11a. The O_2_ concentration in an indoor environment exhibited relatively minor variations of 20.85~21.2% during the measurement. In outdoor measurement, the sensor system was mounted on a cart and placed outside the Optoelectronic Science and Technology Laboratory. The outdoor detection experiments were conducted on 9 and 10 April, each lasting for a period of seven hours, from 11 a.m. to 6 p.m. The results of these experiments can be found in Figure 11b,c, respectively. Compared with indoor monitoring, larger fluctuations in concentration levels were observed in the outdoor monitoring of O_2_.

## 6. Conclusions

In this paper, an O_2_ sensor system using multi-pass absorption spectroscopy and WMS-2*f*/1*f* technology was developed. A DFB laser with butterfly packaging was used to target the O_2_ absorption line located at 760.89 nm. A compact Herriott cell was used which can provide an effective optical path of 3.3 m. The optimum modulation amplitude was obtained, and sensor performance was evaluated by experiments. Based on Allan variance analysis, the MDL is 0.054% under 1 s integration time, which can be improved to 0.009% when the integration time reaches ~60 s. Table 1 listed the performance and parameters of O_2_ detection systems based on TDLAS technology in recent years. Upon comparison, it is evident that the system exhibits high sensitivity. Additionally, the dual-channel orthogonal lock-in amplifier, which was designed using LabVIEW, has effectively reduced the sensor system’s size and weight. This has made the system smaller, enabling easy measurement of O_2_ concentration in outdoor environments. Atmospheric O_2_ monitoring in indoor and outdoor environment on the Beijing Jiaotong University campus was conducted, and the results show that the designed system has good stability, anti-interference ability, and the ability to meet the requirements of long-term on-line monitoring of O_2_ in the environment.

## Figures and Tables

**Table 1:** Comparison of O_2_ detection systems based on TDLAS technology.

Reference	Light Source	Absorption Line	Optical Path	MDL
[16]	VCSEL	764.38 nm	cubic diffuse integrating cavity	350 ppmv
[21]	DFB	760.241 nm	56 cm	6500 ppmv
[29]	VCSEL	~760 nm	~10 cm	1000 ppmv
[30]	DFB	~760 nm	~4.3 m	1000 ppmv
This paper	DFB	760.89 nm	3.3 m	540 ppmv at 1 s integration time and 90 ppmv at ~60s integration time

## Data Availability

The data presented in this study are available on request from the corresponding author.
